# Gene Therapy in Inherited Retinal Diseases: An Update on Current State of the Art

**DOI:** 10.3389/fmed.2021.750586

**Published:** 2021-10-15

**Authors:** Alessia Amato, Alessandro Arrigo, Emanuela Aragona, Maria Pia Manitto, Andrea Saladino, Francesco Bandello, Maurizio Battaglia Parodi

**Affiliations:** Department of Ophthalmology, Scientific Institute San Raffaele Hospital, Milan, Italy

**Keywords:** inherited retinal dystrophies, gene therapy, Stargardt disease, retinitis pigmentosa, choroideremia, X-linked retinoschisis

## Abstract

**Background:** Gene therapy cannot be yet considered a far perspective, but a tangible therapeutic option in the field of retinal diseases. Although still confined in experimental settings, the preliminary results are promising and provide an overall scenario suggesting that we are not so far from the application of gene therapy in clinical settings. The main aim of this review is to provide a complete and updated overview of the current state of the art and of the future perspectives of gene therapy applied on retinal diseases.

**Methods:** We carefully revised the entire literature to report all the relevant findings related to the experimental procedures and the future scenarios of gene therapy applied in retinal diseases. A clinical background and a detailed description of the genetic features of each retinal disease included are also reported.

**Results:** The current literature strongly support the hope of gene therapy options developed for retinal diseases. Although being considered in advanced stages of investigation for some retinal diseases, such as choroideremia (CHM), retinitis pigmentosa (RP), and Leber's congenital amaurosis (LCA), gene therapy is still quite far from a tangible application in clinical practice for other retinal diseases.

**Conclusions:** Gene therapy is an extremely promising therapeutic tool for retinal diseases. The experimental data reported in this review offer a strong hope that gene therapy will be effectively available in clinical practice in the next years.

## Introduction

Inherited retinal diseases (IRDs), also referred to as inherited retinal dystrophies, are a clinically and genetically heterogeneous group of neurodegenerative disorders, primarily involving photoreceptors, retinal pigment epithelium (RPE), and/or the choroid. Taken as a whole, IRDs have an estimated global prevalence of about 1 in 2,000 individuals, affecting more than two million people worldwide, and standing out as the leading cause of blindness in the Western working-age population (1).

Inherited Retinal Diseases are classified according to different criteria, including the primarily diseased retinal cell type (rod-dominated disease, cone-dominated disease, generalized retinal degenerations, and vitreoretinal disorders), the age of onset, the progression of visual impairment over time (stationary or progressive), and the presence or absence of associated systemic features (isolated or syndromic IRDs).

Since the identification of the first gene responsible for an IRD back in 1988 (2), enormous progress has been made in the field of molecular testing, leading to the identification of over 270 disease-causing genes (https://sph.uth.edu/retnet/sum-dis.htm).

Nevertheless, until very recent times, these major diagnostic advances did not go hand in hand with the development of vision-sparing or vision-restoring therapeutic strategies and IRDs have been long accounted as largely incurable diseases.

Over the last decades, this view has changed, as novel therapeutic options started to be explored in preclinical studies, with some of them transitioning to the clinical setting, including gene therapy, cell therapy (3), retinal prosthetics (4), and even direct brain stimulation (5).

In this context, gene-based therapies stand out as one of the most promising frontiers of IRD treatment and the introduction of voretigene neparvovec-rzyl (Luxturna), the first FDA- and EMA-approved gene therapy treatment, paved the way for further research.

The first section of this review is aimed at making the reader familiar with the basic concepts and nomenclature used in the field in retinal gene therapy, while the second section explores in detail those IRDs for which gene-based therapy approaches have made it to the human trial stage. Both sections adopt a combined descriptive and analytic approach, in order to provide a broad overview of the state of the art of gene therapy in IRDs, including discussion of current obstacles and research gaps, as well as a description of the most promising strategies that are being developed to overcome these obstacles and to fill these gaps.

## Methods

We searched all English language and human subject articles using keywords search of MEDLINE library. The keywords included the following: Inherited retinal dystrophies; gene therapy; Stargardt disease (STGD); Retinitis pigmentosa (RP); chorioderemia; X-linked retinoschisis (XLRS); Leber's congenital amaurosis (LCA). All the references were carefully examined by two expert researchers (Alessandro Arrigo, Alessia Amato) which collected and ordered all the relevant information, considering the main topic of this review as expressed in the manuscript title.

## Section 1: Basics Concepts in Retinal Gene Therapy

Gene therapy is the treatment of a disease through genetic material (DNA or RNA), that is transferred into the cells of the patient in order to modify gene expression. Since 1990, when the first gene therapy trial was performed in two children with adenosine deaminase (ADA)-deficient severe combined immunodeficiency (SCID) (6), this approach has been studied for and applied to a variety of inherited and acquired disorders, with more than 20 gene therapies officially approved for clinical uses by the drug regulatory agencies from different countries.

### The Eye as an Ideal Target for Gene Therapy

Since the dawn of gene therapy, the human eye has always presented itself as an appealing target for a number of reasons.

First, owing to the presence of the so-called blood-retinal barrier (BRB), made up of the tight junctions between the endothelial cells of retinal microvasculature (i.e., inner BRB) and between RPE cells (i.e., outer BRB), the retina is an immune-privileged site, meaning that the introduction of foreign substances is less likely to cause an inflammatory reaction.

Second, the eye is a relatively small and enclosed compartment, which in turn has two important implications: lower doses of therapeutics are needed and the risk of systemic dissemination of the locally administered vector is generally negligible (which, again, minimizes the risk of immune responses).

Moreover, since they are paired organs, it is possible to treat one eye and use the fellow eye as an ideal control to assess the efficacy and safety of the treatment.

Finally, the eye is an easily accessible district, from both a surgical [via subretinal or intravitreal injections (IVIs)] and a diagnostic standpoint, so that non-invasive studies can be performed to monitor function and structure of the treated retinas.

### Gene Delivery Systems

With regards to gene therapy, it is crucial to differentiate between *ex vivo* approaches, where patients' cells are collected, cultured, modified, and transplanted back to the same individual (7), and *in vivo* approaches, where a gene-therapy vector is directly administered to a living organism. Though some attempts are being made in the preclinical setting with transplant of gene corrected cells (8), ocular gene therapy relies on an *in vivo* approach, since the genetic material is administered directly into the patient's eye by means of a subretinal or IVI.

Another important distinction is in the way nucleic acids are delivered to their target cells. DNA and RNA are, in fact, large in size and negatively charged molecules, two features that hinder their ability to cross cell membranes. This obstacle is overcome by employing a variety of gene delivery systems, which can be divided into two main categories: *non-viral* and *viral* systems.

#### Viral Delivery Systems

Viruses are the most used vectors and the process by which they infect and release their genetic content into target cells is termed transduction. Several different recombinant viruses, that are replication deficient, can be used to deliver therapeutic nucleic acids, with differences in terms of cargo limits, integration capabilities, transduction efficiency, cellular tropism, and risk of immune responses.

Adenoviruses (Ads) are a family of DNA viruses that can infect quiescent and dividing cells and replicate in the host nucleus without integrating their genome. Adenoviruses have been largely tested as gene therapy vectors, mainly due to their cargo capacity (approximately 8–36 kb) and ability to transduce many cell types. As far as IRDs are concerned, however, conventional Adenoviral vectors (AVs), constructed by substituting the E1 region with the transgene cassette of interest (9), had limited success, owing to the expression of some viral genes in the infected target cells, which enhanced immunogenicity and undermined treatment longevity, even in the immune-privileged environment of the human eye. These issues have been partially addressed with second- and third-generation vectors, characterized by progressive stripping of all viral coding sequences and implementation of helper-dependent AVs. However, problems with contaminating helper viruses, vector instability, and replication-competent AVs have been reported (10, 11).

Adeno-Associated Viruses (AAVs) are defective single-strand (ss) DNA parvoviruses with more than 20 integration sites in the human genome. Recombinant AAVs (rAAVs) vectors are by far the most frequently used ones in gene therapy approaches for IRDs, because of their lack of pathogenicity, favorable immunologic profile (since, unlike AVs, they do not carry any virus open reading frame), non-integrating nature in the absence of rep protein (which minimizes the risk of insertional mutagenesis, unlike LVs), ability to provide a stable transgene expression and extended retinal tropism. To date, 13 naturally occurring serotypes of AAV have been isolated from primates (AAV1–AAV13): different serotypes have a different capsid conformation and different properties, especially as far as tropism is concerned. Moreover, AAVs can be modified in several ways, for example by packaging the viral genome bordering the transgene into the capsid of a different AAV serotype, process known as pseudotyping or cross-packaging (e.g., an AAV2/8 vector is a pseudotype in which the genome of AAV2 serotype is packaged into an AAV8 capsid) (12). Both serotype and pseudotype choice are important to optimize vector design for the target disease. To date, the serotypes and pseudotypes that have been used in clinical trials for IRDs include AAV2/5, AAV2/8, and AAV8. The major disadvantage of rAAV vectors is their limited cargo capacity, which cannot exceed 4.7 kb. Although with an apparently reduced photoreceptor transduction efficiency, dual AAV vectors—each of which contains half of a large transgene expression cassette—have been shown to improve retinal phenotype in murine models of IRDs (13–15).

Lentiviruses (LVs) are retroviruses with a larger packing capacity (8 kb), which makes them a compelling alternative to AAV vectors for those IRDs whose causative gene coding sequence exceeds the 4.7 kb limit, such as ABCA4-related Stargardt's disease and MYO7A-related Usher's syndrome type 1B. So far, the retroviral variant of human immunodeficiency virus type 1 (HIV-1) and the equine infectious anemia virus (EIAV) have been studied for IRDs. Lentivirus vectors have two main drawbacks. First, LVs are integrating in nature and genomic integration, if on the one hand leads to a sustained expression of the foreign DNA, on the other hand carries a risk of insertional mutagenesis (16, 17). Such risk may not be justified in the case of IRDs, since in post-mitotic tissues, like the retina, a stable transduction can be achieved even by lentiviral episomes. This limitation can be overcome by employing integration-deficient lentiviral vectors (IDLVs), which have been successfully used in a rodent model of retinal degeneration (18). Second, LVs are capable of effectively transducing RPE cells and only to a lesser extent, which is generally insufficient for therapeutic purposes, differentiated photoreceptors (19, 20).

#### Non-viral Delivery Systems

Non-viral delivery systems have some advantages over viral delivery systems, including potentially unlimited cargo capacity, simultaneous conveyance of multiple therapeutics, low immunogenicity, and inexpensive manufacturing procedures.

Non-viral delivery systems use physicochemical agents to compact the DNA and/or transport it across the membranes.

Physical methods include, for instance, sonoporation (21), and electroporation (22) (i.e., the use of ultrasound or electricity to temporarily increase cell permeability) and direct injection of DNA into target cells (23), respectively, offering no protection from enzymatic degradation of therapeutic nucleic acids.

Chemical agents, which protect the payload from the action of nucleases, include, among others, cationic liposomes, lipopolyplex, and nanoparticles (NPs) (24–27).

Though some preclinical gene therapy studies have successfully used non-viral DNA systems (28–30), these agents are hard to export to an *in vivo* clinical setting, mainly because of transiency of gene expression (31, 32), ultimately resulting in a relatively inefficient delivery (9).

### Administration Routes

At least in part, the success of gene therapy approach depends on its administration route. So far, the ongoing clinical trials for IRDs have relied on two injection modalities (i.e., subretinal and intravitreal), both of which have their strengths and weaknesses.

Subretinal injection (SRI) is adopted in most clinical trials, since it allows for the administration of the vector in close proximity to the most common cell target site (i.e., RPE and photoreceptors). Moreover, SRI places the therapeutic material in a closed immune-privileged compartment, thus diminishing the risk of immune reactions. Of course, SRI is a delicate procedure, requiring a vitrectomy, retinotomy, and the development of a transitory iatrogenic neuroretinal displacement and it is potentially associated with a number of complications, including retinal tears, cataract progression, or retinal/choroidal hemorrhages. With respect to SRI, vitreoretinal subspecialists have reported the utility of *in vivo* real-time monitor of the surgical act through integrated optical coherence tomography (OCT) operating microscope (33).

Intravitreal injection is certainly less invasive and technically challenging and can be performed in a clinic setting, thereby extending the accessibility of gene-based therapies to larger populations. However, while adequately transducing inner retinal cells, such as retinal ganglion cells (RGCs), the intravitreally-administered vectors are far less effective on outer retinal layers, due to dilution in the vitreous cavity and to the inner limiting membrane (ILM) barrier, which is particularly thick in primates. Therefore, in order to compensate for these obstacles and observe a significant therapeutic effect on target cells, much higher doses would have to be injected in a non-immune-privileged site. This, in turn, brings about a significant risk of immunogenicity, not only in the form of potential adverse inflammatory reactions, but also in the form of neutralizing antibodies, accounting for the need of repeated injections in intrinsically frail retinas.

Apart from these two main administration routes, a third one is currently being studied, that is suprachoroidal delivery, whereby therapeutics are conveyed in the space located between the sclera and the choroid. Though preclinical studies and clinical trials showed a good safety, comparable to that of IVIs, spreading of vectors into the systemic circulation is a potential risk (34–37). Finally, preclinical studies have attempted a sub- ILM approach (38).

### Gene-Based Therapies in IRDS: the Strategies

Gene-based therapies can rely on different strategies, depending on the features, and molecular pathogenesis of the diseases being addressed, which can be schematically divided into two main categories:

- Autosomal recessive (AR) or X-linked recessive (XLR) diseases; when a disease-causing mutation abrogates the normal gene function, it is defined as loss-of-function mutation. In this case, the mechanism underlying the associated disorder is caused by a loss of function, whereby a single copy of the gene (in case of AR inheritance) or the absence of functional alleles (in case of XLR inheritance in hemizygous males) is not sufficient to guarantee sufficient levels of the protein. The best way to address recessively inherited retinal dystrophies is by gene augmentation (or gene replacement).
- Autosomal dominant (AD) diseases; these diseases are usually caused by gain-of-function mutations, whereby an aberrant protein is formed, resulting in disruption of cellular or tissue activity, or by a dominant negative effect, in which a defective subunit poisons a macromolecular complex. Gene augmentation alone is not enough to address AD IRDs, which require more sophisticated approaches, broadly classified as forms of gene silencing (or knockdown). Gene silencing can be associated with gene replacement, often by means of dual AAV vectors.

#### Gene Augmentation

Since its initial conceptualization back in the 1960s (39), the idea of gene therapy was based on the straightforward assumption that monogenic recessive disorders could be cured by replacing a faulty gene with a normal copy of it delivered through therapeutic vectors. This approach is called gene augmentation (or gene replacement) because the synthesis of the protein is augmented and its function, at least partially, restored. The gene of interest can be delivered as DNA or mRNA. Though having the advantage of not requiring delivery into the nucleus, thereby reducing the risk of integration into the host genome, the mRNA platform is far more immunogenic and less stable than the DNA platform, which is therefore the preferred one for ocular gene therapy (40). Most clinical gene therapy trials, as well as the first, and currently only, FDA- and EMA-approved treatment for an IRD, voretigene neparvovec-rzyl (Luxturna), rely on gene replacement, that does not require modification of native DNA and therefore is particularly compelling, owing to its simple design and relative ease of investigation.

#### Gene Silencing

Gene augmentation is an established approach for recessive monogenic disorders, but it is not suited to AD diseases resulting from pathological gain-of function mutations. In these cases, the therapeutic goal is to prevent the altered gene from expressing and encoding an aberrant protein that would interfere with normal cell function. To do so, it is possible to adopt several different approaches, which, schematically, can act at three levels: DNA, mRNA, and the intermediate process in between them (i.e., transcription).

In all of these cases, the host nucleic acids can be targeted in two ways, that is in a mutation-dependent fashion, whereby specific allele inhibition is sought in order to allow the expression of the wild-type copy of the gene, or in a mutation-independent fashion, in which a combined approach with gene augmentation is mandatory, since both copies of the gene (the mutated and the functional one) are silenced and replaced by a non-silenced wild-type form of it. Although allele-specific strategies do not disrupt the endogenous wild-type genome, allele-independent approaches are more far more practical since they don't have to be customized for specific disease-causing mutations. Allele-independent strategies, however, require the expression of both nucleic acid molecules in the same vector and are therefore limited by packaging issues.

##### DNA-Based Therapies (Genome Editing)

To date, the CRISPR/Cas9 system is considered the most advanced genome editing tool. This system consists of the Cas9 endonuclease, delivered into target cells in conjunction with a guide RNA (gRNA), which is able to cut the genome at any desired genomic location. The double-strand breaks (DSBs) created by the enzyme subsequently activate one of the DNA repair pathways: non-homologous end joining (NHEJ), homology-directed repair (HDR), or microhomology-mediated and joining (40). The development of the homology-independent targeted integration (HITI) strategy, that relies on NHEJ rather than HDR, enabled gene editing in the retina, since post-mitotic cells lack HDR, and was first used in a rat model of MERTK-related RP, with structural and functional improvements (41).

Other than for strict genome editing, the CRISPR/Cas9 can also be used as part of a gene silencing strategy to inactivate mutant alleles causing a toxic gain-of-function, or as part of a splicing modulation approach to prevent the inclusion of pseudoexons (i.e., deep-intronic sequences erroneously recognized as exons due to DNA mutations) that would result in the synthesis of an aberrant protein. The CRISPR/Cas9-based transcript degradation approach has been successfully used in studies on autosomal dominant retinitis pigmentosa (adRP) associated with rhodopsin mutations (RHO-adRP) (42–45), while the latter strategy of restoring splice defects has been applied to a deep intronic mutation of CEP290 in preclinical models (46).

##### mRNA Silencing

mRNA silencing strategies rely on a variety of antisense inhibitors (i.e., nucleic acid molecules that are complementary to and hybridize with protein-coding mRNAs) and function either by clearing mRNA molecules or by repressing their translation. mRNA silencing strategies include:

###### Ribozymes.

Ribozymes are RNA molecules able to catalyze a chemical reaction in the absence of proteins, which can be used to promote site-specific cleavage of a target phosphodiester bond in order to inhibit gene expression (47). Ribozymes have been the first RNA-based therapeutic strategy investigated in IRDs (48). This approach, however, has been largely abandoned and replaced by newer RNA-based technologies, mainly because the recognition sequence of these molecules are highly represented in the human genome, with a consequent risk of off-target effects, and because of the computational complexity of identifying mRNA cleavage sites (49).

###### Small Interfering RNAs.

RNA interference (RNAi) is another post-transcriptional gene silencing technology based on an evolutionary conserved pathway (50, 51). The effectors of RNAi, called small interfering RNAs (siRNAs), are 21–23 nucleotide-long double-stranded (ds) RNA molecules able to inhibit gene expression by binding to specific mRNAs. These siRNAs can be either naturally obtained in the presence of long pieces of dsRNA, which—for gene therapy purposes—are delivered by DNA vectors and cleaved by an RNase III enzyme called Dicer (52), or can be synthetically produced and directly introduced into the cells (51), the latter approach being less immunogenic, since long ds-RNAs can trigger an innate immune response (53). Unlike ribozymes, whose nucleolytic activity is independent of proteins, siRNAs do not directly take part to complementary mRNA degradation. Instead, siRNAs, once in the cytoplasm of the target cell, are incorporated in the so-called RNAi-induced silencing complex (RISC), which contains both the helicase that unwinds the ds-siRNA into its sense and antisense strands and the endonuclease Argonaute-2. The latter enzyme is in charge of clearing the sense strand of the siRNA molecule and the target mRNA sequence, the access to which is guided by the complementarity to the antisense strand (54). Just like with ribozymes, off-target effects are a major obstacle in the translation to the human clinics, since they can induce a toxic phenotype in target cells, especially in the presence of specific motifs (55); new computational methods able to screen candidate siRNAs can help overcome such obstacles (56). Another possible side effect is the elicitation of immune responses, which is more likely to occur when certain sequences are present (57). Both the above-described post-transcriptional silencing strategies (i.e., ribozymes and RNAi) have been successfully used in animal models of IRDs, with particular reference to adRP associated with rhodopsin mutations (58–61), providing proof-of-concept for RNA-based retinal gene therapies.

###### Antisense Oligonucleotides.

Antisense oligonucleotides (AONs) are small DNA or RNA molecules that can be designed complementary to their target mRNAs (62). Over the last years, AONs have been the object of increasing interest among retinal gene therapists, since they represent the only approach, other than gene augmentation, that has made it to the clinical setting (see section 2: Gene therapy in IRDS).

Depending on their chemical properties, AONs can display two distinct mechanisms.

First, they can act as authentic gene silencers, by mediating, similarly to siRNAs, degradation of target transcripts in a RNase H1-dependent fashion (63). This mechanism has been studied *in vitro* for a NR2E3 variant underlying adRP (64) and *in vivo* for RHO-adRP, both in the preclinical and clinical settings (65).

Other than for knockdown purposes, AONs can also be used as pre-mRNA splicing modulators, an interesting application in the field of IRDs, since up to 15% of all retinal degeneration-causing mutations affect the splicing machinery (66). In this context, AONs generally promote exon skipping, meaning that they bind to target pre-mRNA sequences, and block the recruitment of splicing factors. This approach proves particularly useful when exclusion of pseudoexons is sought, as in the case of CEP290-related LCA (see section 2: Gene therapy in IRDS), which at present stands out as the most advanced application of this technology, having reached phase III of clinical evaluation (NCT03913143). Though promising proof-of-concept studies have been developed for many other genes, such as OPA1, CHM and ABCA4 (67–72), the only other IRD-causing gene whereby the AON-based approach has been translated to the clinics is USH2A (73), for which—following the success of the phase 1/2 trial (NCT03913143)—two final stage registration trials are planned to start by the end of 2021.

Despite the unquestionable advantages of RNA therapeutics over other gene-based strategies, such as the titratability and affordability of the employed molecules, the reversibility of their effects, and the non-genome altering approach, some major challenges still lie ahead of this field. One of such challenges is related to the instability of naked nucleic acids, which are promptly degraded by endonucleases (74), resulting in a time-limited effect. To avoid enzymatic clearance of antisense molecules, two strategies can be adopted. The first strategy is chemical modification of the nucleic acids, in order to make them endonuclease-resistant, as it has been done with both siRNAs and with first-, second-, and third- AONs (75–80). The second approach is to package these therapeutic molecules inside vectors, either viral (81), thus requiring a SRI, or non-viral (82), allowing for repeated, though sufficiently long-lasting, IVIs. As a matter of fact, in a recent study on a mouse model of CEP290-LCA, IVI injection of second-generation AONs compared to SRI of AAV-packaged AONs, while exhibiting comparable duration of effect (approximately 1 month), turned out to be more effective (81). This is probably due to the fact that chemically modified AONs are more efficiently taken up by cells compared to AAVs, further underscoring the importance of developing IVI-based approaches.

##### Transcriptional Silencing

Therapeutic strategies for gain-of-function mutations mainly rely on DNA-based technologies, such as the CRISPR/Cas system, and on RNA silencing, thereby acting upstream and downstream of the transcription process.

Over the last decade, efforts have been made to target the intermediate step between DNA and RNA, that is the process by which genetic sequences are used as templates to assembly pre-mRNA transcripts.

In the field of IRDs, three different mutation-independent transcriptional repression strategies have been developed and successfully applied to preclinical models of RHO-adRP.

The first of these strategies employed zinc finger-based artificial transcription factors (ZF-ATFs) targeting the human rhodopsin promoter to achieve, in a mouse model of adRP, transcriptional silencing of both wild-type and mutated RHO alleles in a mutation-independent fashion, which was followed by gene replacement of the endogenous RHO copies (83).

The same group showed that *in vivo* AAV-mediated delivery of a modified version of a synthetic transcription factor (TF), uncoupled from its repressor domain and designed to bind a 20-bp DNA sequence motif (ZF6-cis sequence), could block RHO expression in living porcine retina without significant genome-wide transcript perturbations (84).

Based on these results, the authors went on to further unveil the potential of TF-based transcription silencing, this time by delivering to rods an ectopic TF (i.e., a TF which is normally not expressed by rods) with a DNA-binding preference for the ZF6-cis sequence photoreceptors of pigs, resulting in suppression of RHO with limited off-target effects in a mouse and porcine retinas (85).

Taken together, these data support the role of transcriptional silencing as a promising novel mode to treat gain-of-function mutations in autosomal dominantly inherited IRDs.

#### Non-targeted Gene Therapies

With over 270 genes associated with IRDs, developing mutation-specific or even gene-specific approaches becomes challenging. In this context, the role of non-targeted gene therapy is to provide alternative strategies, aimed at improving vision independently of the causative gene (86). Attempts have been made by delivering via AAV vectors molecules capable of prolonging photoreceptor cell survival, including neurotrophic factors, such as ciliary neurotrophic factor (CNTF), glial cell line-derived neurotrophic factor, basic fibroblast growth factor and rod-derived con viability factor, and anti-apoptotic agents, such as X-linked inhibitor of apoptosis (87–92). Other than CNF, whose efficacy was rather modest (93), none of these approaches have been tested in humans so far.

As an alternative mutation-independent strategy, optogenetics delivered as non-targeted gene therapy for advanced RP is also being tested. In this case, the assumption is that inducing expression of light sensitive opsins in bipolar or RGCs could activate the visual pathway even in the absence of viable photoreceptors (94–97). There are three ongoing clinical trials using optogenetics in RP patients, RST-001 (NCT02556736), GS030 (NCT03326336), and BS01 (NCT04278131), while one phase 1/2 clinical trial, vMCO-I, has been recently completed (NCT04919473).

## Section 2: Gene Therapy in IRDS

### Gene Therapy in Leber Congenital Amaurosis

Leber congenital amaurosis represents a group of IRDs, with an estimated prevalence of 2–3/100,000, characterized by four clinical milestones: severe and early visual impairment (usually occurring by the 6^th^ week of life), sensory nystagmus (an indirect manifestation of the low fixation ability), amaurotic pupils (an expression of the poor retinal sensitivity from the retina to the brainstem), and non-recordable electroretinography (ERG) responses (98). Other frequent phenotypic features include high refractive errors, photophobia, nyctalopia, and the so-called oculodigital sign of Franceschetti, consisting of a repetitive, deep rubbing of the eyes. Association between LCA and keratoconus and cataracts, as well as with a wide range of systemic manifestations, including intellectual disability, olfactory dysfunction, stereotypical movements, and behaviors has also been reported (98–102). From a clinical standpoint, LCA exhibits an extremely heterogeneous phenotype, ranging from an essentially normal retina to variable degree of vessel attenuation, bone spicule pigmentation, pseudopapilledema, macular coloboma, salt and pepper pigmentation, yellow confluent peripheral spots, white retinal spots, preserved para-arteriolar RPE (PPRPE) and Coats reaction, with some gene-specific features (98–105).

A milder form of the same disease spectrum has been described using several different expressions, including early-onset severe retinal dystrophy (EOSRD), severe early childhood-onset retinal dystrophy (SECORD) and early-onset RP (106). Unlike LCA, which is present at birth or within the first weeks of life and is associated with nystagmus, poor pupillary responses and abolished ERGs, EOSRD/SECORD has a slightly later onset (after infancy but before 5 years of age) and is characterized by a better residual visual function and ERG responses (106). Nevertheless, large genotypic overlap exists between these two disease entities, though certain genes are more frequently associated with LCA and others with EOSRD/SECORD (106).

#### Genetic Features

Leber congenital amaurosis is mostly inherited in an AR fashion, though for some genes, like CRX, AD patterns have been reported (107). So far, more than 25 genes, overall accounting for at least 80% of all LCA cases, have been described, the most common of which are listed in Table 1 (106–126). Accordingly with the current literature, the most common LCA-causing genes are, in descending order, *GUCY2D, CEP290, CRB1, RDH12*, and *RPE65* (1, 3, 4). LCA-associated genes encode proteins, whose functions can be divided into four main categories: phototransduction (e.g., *GUCY2D*), photoreceptor morphogenesis (e.g., *CRB1* and *CRX*), retinoid cycle (e.g., *RDH12* and *RPE65*), and ciliary transport processes (e.g., *CEP290*) (98).

**Table 1:** Main genes associated with the onset of LCA.

**Gene**	**Protein**	**Function**	**Molecular weight (kDa)**	**Reference**
*IMPDH1*	Inosine 5′-monophosphate dehydrogenase 1	Guanine synthesis	~55	(5)
*CRX*	Cone–rod homeobox	Photoreceptor morphogenesis	~32	(6)
*CRB1*	Crumbs homolog 1	Photoreceptor morphogenesis	~154	(7)
*GDF6*	Growth differentiation factor 6	Photoreceptor morphogenesis	~14	(8)
*SPATA7*	Spermatogenesis-associated protein 7	Photoreceptor ciliary transport	~68	(9)
*LCA5*	Libercilin	Photoreceptor ciliary transport	~80	(10)
*RPGRIP1*	Retinitis pigmentosa GTPase regulator-interacting protein 1	Photoreceptor ciliary transport	~147	(11)
*CEP290*	Tubby-like protein	Photoreceptor ciliary transport	~290	(12)
*TULP1*	Tubby-like protein	Photoreceptor ciliary transport	~70	(13)
*CLUAP1*	Clusterin associated protein 1	Photoreceptor ciliary transport	~48	(14)
*IQCB1*	Intraflagellar transport 140 chlamydomonas homolog protein	Photoreceptor ciliary transport	~69	(15)
*IFT140*	Intraflagellar transport 140 chlamydomonas homolog protein	Photoreceptor ciliary transport	~165	(16)
*ALMS1*	ALMS Protein	Photoreceptor ciliary transport	~460	(17)
*GUCY2D*	Guanylate cyclase-1	Phototransduction	~120	(18)
*AIPL1*	Aryl-hydrocarbon-interacting-protein-like 1	Phototransduction/protein biosynthesis	~43	(19)
*RD3*	Protein RD3	Protein trafficking	~70	(20)
*RPE65*	Retinoid isomerase	Retinoid cycle	~65	(21)
*RDH12*	Retinol dehydrogenase 12	Retinoid cycle	~38	(22)
*LRAT*	Lecithin:retinol acyl transferase	Retinoid cycle	~25	(23)

#### Gene Therapy in RPE65-LCA

The *RPE65* gene product plays a critical role in the retinoid cycle, so that *RPE65* mutations affect visual function before photoreceptor structure. Therefore, in contrast with many other IRDs in which visual dysfunction results from rods and cones death, RPE65-LCA patients retain viable cells for years before significant degeneration becomes evident. This structure-function dissociation makes RPE65-related retinal dystrophies a particularly compelling target for gene replacement strategies.

Proof of principle for retinal gene therapy came from the pioneering studies conducted in the early 2000's on a peculiar canine model (127, 128). These preclinical studies employed a subset of Briard dogs with a homozygous 4-bp deletion in the *RPE65* gene resulting in a premature stop codon, thereby appearing to be an excellent spontaneous model for human RPE65-related LCA (129). These studies reported, after a single SRI of AAV-mediated *RE65*, an improvement in blue light stimulated dark-adapted ERGs and cone flicker, pupillometry, and VEP in the injected eyes and in qualitative behavioral assessments in the treated dogs, which were stable 3 years after the procedure (127, 128). Further evidence of the efficacy of this approach came from the naturally *Rpe65*-mutated rd12 murine model and from the genetically built *Rpe65*^−/−^ knockout mouse (130, 131). Following the success of animal studies, clinical trials were initiated in 2007 by groups from the UK and the US (132–134), culminating in the first FDA- and EMA-approved AAV-based retinal gene therapy drug, voretigene neparvovec-rzyl (Luxturna) (135). Follow-up studies revealed stable improvements in most patients, peaking at 6–12 months after injection (136–138), but observational trials aimed at evaluating the long-term effects of Luxturna are still ongoing (NCT03602820, NCT01208389).

#### Gene Therapy in CEP290-LCA

The protein encoded by the *CEP290* gene localizes to the photoreceptor connecting cilium and, besides microtubule-associated transport across the cilium, is required for outer segment (OS) regeneration and phototrandusction. The most common *CEP290* mutation is the so called IVS26 c.2991+1655A>G mutation (p.Cys998X), an adenine to guanine point mutation located within intron 26 creating a novel splice donor site, which results in the inclusion of a pseudoexon in the mRNA and in the consequent creation of a premature codon stop. This mutation has been addressed by means of two innovative approaches.

The first strategy relies on a CRISPR/Cas9 system, called EDIT-101, consisting of an AAV5 vector used to deliver the *Sthaphylococcus aureus* Cas9 and CEP290-specific gRNAs with no identified off-targets. EDIT-101, or a non-human primate (NHP) surrogate vector, were shown to restore normal splicing *in vitro* (in photoreceptor-containing retinal explants) and *in vivo* (in mice and NHPs) with no serious adverse events (139).

The second strategy exploits the AON technology to remove the 128-bp pseudoexon included in the IVS26-mutated CEP290 mRNA transcript. Preclinical evidence of the efficacy of the AON designed to restore IVS26 splicing defects, called QR-110, came from *in vitro* studies on LCA10 fibroblasts (140).

In the wake of these results, IVI of this oligonucleotide was successfully attempted in NHP s (141), finally reaching the clinical setting with a phase I/II trial showing vision improvement at 3 months with no complications in LCA type10 patients treated with multiple doses of intravitreal QR-110 (142). A phase II/III multiple-dose clinical trial is still ongoing and is aimed at evaluating efficacy, safety, tolerability, and systemic exposure of QR-110 administered via IVI in patients with LCA type 10 due to *CEP290* c.2991+1655A>G mutation after 24 months of treatment (NCT03913143).

#### Gene Therapy in Other Forms of LCA

Although so far only two LCA-associated genes have made it to the human trial stage, for many other disease-causing genes, including *GUCY2D, CRB1*, and *RDH12*, preclinical studies are underway, showing promising results (143–148).

### Gene Therapy in Retinitis Pigmentosa

**Figure 1:**
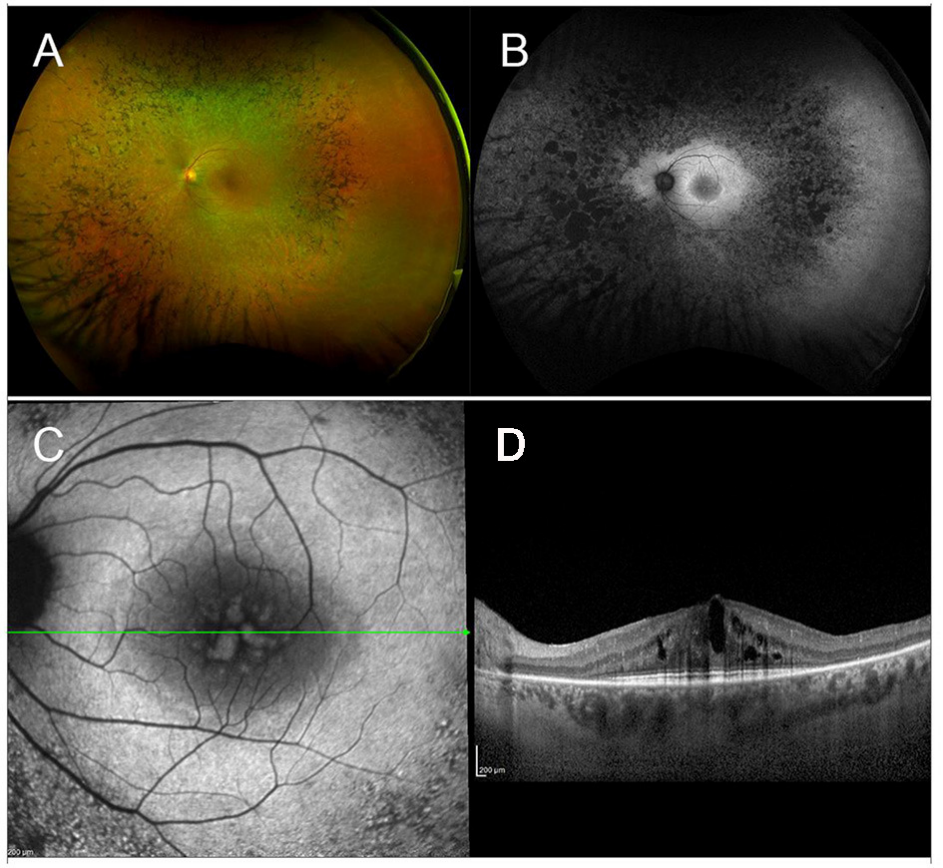
Multimodal imaging in RP. **(A)** Ultra-wide field retinography displays the typical triad: optic disc pallor, vessel attenuation, and bone spicule pigmentation. **(B)** Fundus autofluorescence shows the typical perifoveal ring of hyperautofluorescence and multiple hypoautofluorescent regions, corresponding to the pigment deposits, and to the areas of RPE atrophy. **(C)** Blue fundus autofluorescence shows a petaloid hyperautofluorescent pattern, compatible with a cystoid macular edema. **(D)** Structural OCT confirms the presence of intraretinal cysts.

The term RP refers to a heterogeneous group of IRDs, with variable phenotypes—ranging from mild nyctalopia to total blindness—and genotypes—with over 100 identified RP-causing genes. All inheritance patterns are possible, including AD, AR, or X-linked disorders, whereas maternal (mitochondrial) inheritance is very rare in RP (149). The estimated prevalence of this multiform condition is 1 in 3,000–7,000 individuals (149, 150). In the initial stages, rod photoreceptors degenerate, resulting in night blindness, with difficulty seeing in dim light and adapting to changes in light sensitivity, and in visual field (VF) constriction, starting from the mid-periphery and extending toward the center, eventually leading to complete loss of peripheral vision, the so-called “tunnel vision” (151). With disease progression, also cones become affected and visual acuity (VA) declines. From a clinical standpoint, the fundus appearance of RP features a typical triad, consisting of attenuated retinal vessels, intraretinal pigment deposits with a bone spicule configuration, and optic disc pallor (Figure 1).

Though ERG has been long used to help diagnose and monitor RP, nowadays multimodal imaging is of crucial importance for both initial assessment and follow-up of RP patients. Fundus autofluorescence (FAF), shows a coexistence of hypoautofluorescent regions (correlated to the masking effect of pigment deposits or to the presence of areas of RPE atrophy) and hyperautofluorescent regions (usually in the form of an hyperautofluorescent perifoveal ring) (152, 153). Optical Coherence Tomography shows decreased thickness of the outer nuclear layer (ONL) and loss of external limiting membrane (ELM) and ellipsoid zone (EZ), all of which were shown to correlate well with VF defects (154, 155). More recently, a novel imaging technique, optical coherence tomography angiography (OCTA), has been implemented to explore the existence and potential clinical relevance of different retinal and choroidal vascular patterns in RP patients (156, 157).

Considering the heterogeneity of RP, specific genetic features and currently ongoing clinical trials will be discussed separately for each relevant RP-associated gene.

#### RHO-RP

##### Genetic Features

The rhodopsin (RHO) gene was the first identified RP-causing gene (158, 159). Human RHO is a 6.7 kb-long DNA sequence, containing five exons and mapping on the long arm of chromosome 3 (3q22.1). It encodes rhodopsin, a 348-aa light-sensitive G-protein coupled receptor (GPCR) expressed from rod OSs disks. Rhodopsin is the protein that initiates the phototransduction cascade upon absorption of photons by its chromophore, 11-cis retinal. The vast majority of RHO mutations show an AD inheritance pattern (RHO-adRP), accounting for 25% of adRP cases and leading to RP with a toxic gain-of-function or a dominant-negative effect of the mutated protein (160). However, few recessively inherited mutations are described and have been reported to cause a milder phenotype (161).

##### Gene Therapy

As previously discussed (see Section 1: Basics concepts in retinal gene therapy), IRDs related to a toxic gain-of-function cannot be treated with a gene replacement approach. In these cases, in fact, there is a double therapeutic goal of silencing the mutant allele and increasing the wild type to mutant gene expression ratio. These goals can be achieved in an allele-specific or in a mutation-independent fashion, both of which have their pros and cons (see Section 1: Basics concepts in retinal gene therapy). In Section 1: Basics concepts in retinal gene therapy, we provided an overview of the current gene therapy strategies, and we described the three main targets of gene silencing approaches, all of which have been investigated in RHO-adRP, as reported in the excellent paper by Meng et al. (162).

As far as DNA-based therapies are concerned, the CRISPR/Cas9 system technology has been successfully applied to animal models and human retinal explants of RHO-adRP, both in an allele-specific (43–45) and in a mutation-independent way (42, 163).

Post-transcriptional RNA-based silencing strategies have perhaps been the most promising for RHO-adRP.

A dual vector short hairpin RNA (shRNA) suppression and replacement therapeutic strategy for RHO-adRP, named RHONova, proved to restore function and preserve morphology in a murine model of the disease independently of the mutation and received orphan drug designation in Europe and in the US, although there has been no publicly available updates on its clinical development (162–166). More recently, another RNAi-based mutation-independent strategy has been attempted, this time by means of a single AAV2/5 vector expressing both a shRNA targeting human RHO and a healthy copy of the gene, modified so as to be shRNA-resistant, with encouraging morpho-functional results (167). Further preclinical studies are currently being conducted on this gene therapy product candidate, now called IC-100, with a phase 1/2 clinical trial expected to begin by the end of 2021.

Antisense oligonucleotides have been used to promote allele-specific knock-down of P23H-mutant mRNA in a murine model of RHO-adRP, without affecting wild-type RHO expression. This approach yielded excellent preclinical results and transitioned to the clinical stage, with a phase 1/2 clinical trial currently ongoing and is scheduled to conclude in October 2021.

Finally, transcriptional repression strategies have also been attempted in the preclinical setting (83–85), as reported in Section 1: Basics concepts in retinal gene therapy of this review.

#### RPGR-XLRP

##### Genetic Features

X-linked retinitis pigmentosa (XLRP) is responsible for 5–20% of all RP cases. So far, three disease-causing genes have been identified: RP GTPase Regulator (*RPGR*) at the RP3 locus, retinitis pigmentosa 2 (*RP2*) at the RP2 locus and the oral-facial-digital syndrome type 1 (*OFD1*) (168–170).

The *RPGR* gene, whose mutations account for 70–90% of XLRP cases, encodes *RPGR*, a key protein in photoreceptor ciliary function. *RPGR* transcripts undergo a complex splicing process and generate constitutive variants, expressed by most tissues, and *ORF15* variants, which are highly specific for the retina, by using alternative polyadenylation sites and splicing sites (171, 172). While mutations in the exons unique to the constitutive variant are almost exclusive of XLRP, mutations in the *ORF15* exon, considered a mutational hot spot, are also found in cone dystrophy (COD) and cone-rod dystrophy (CORD) pedigrees (173).

X-linked retinitis pigmentosa is regarded as the most aggressive genetic subtype of RP, with hemizygous males exhibiting a particularly severe phenotype, characterized by early onset and rapid progression, eventually resulting in legal blindness by the end of the third decade of life. Heterozygous female carriers usually show some degree of fundus and FAF alterations, with an associated visual function that can range from 20/20 BCVA to no light perception (174–178). The variable extent of retinal involvement in female carriers could be explained by the dominant nature of some *RPGR* mutations or could be the result of a random skewed X inactivation phenomenon.

##### Gene Therapy

X-linked retinitis pigmentosa is the IRD with the highest number of ongoing gene therapy clinical trials, all of which rely on a AAV-mediated gene replacement strategy.

Before it was possible to transition to the clinics, *RPGR* canine models of XLRP (XLPRA1 and XLPRA2) treated with subretinal AAV2/5 full-length human RPGRex1-ORF15 provided preclinical evidence of the beneficial effects of this approach (179).

Moving on to the clinical setting, Nightstar Therapeutics/Biogen recently published the initial results at 6 months of its phase 1/2 dose escalation trial (NCT03116113) (180). Eighteen patients divided in six cohorts of three patients received increasing concentrations of AAV8.coRPGR vector (from 5 × 1,010 to 5 × 1,012 gp/ml) by means of a SRI. The primary outcome of the study was safety and initial results showed no significant concerns aside from subretinal inflammation in patients at the higher doses, that resolved after steroid treatment. Moreover, some secondary endpoints suggest sustained reversal of VF loss.

Another ongoing phase 1/2 trial, sponsored by MeiraGTx, is employing a SRI of an AAV2/5 vector as part of an open label, non-randomized, dose-escalation intervention followed by randomized dose confirmation against a control arm (NCT03252847). The same company recently initiated a phase 3 trial (NCT04671433).

Applied Genetic Technologies Corp. is sponsoring a phase 1/2 clinical trial (NCT03316560) to evaluate the safety and efficacy of SRI of rAAV2tYF-GRK1-RPGR and a phase 2/3 trial, which is scheduled to begin in the second half of 2021 (NCT04850118).

Finally, 4D Molecular Therapeutics launched the first clinical trial attempting to treat XLRP through a single intravitreal delivery of 4D-125, a drug product developed for gene therapy, which comprises an AAV capsid variant (4D-R100) carrying a codon-optimized human RPGPR transgene (NCT04517149).

#### PDE6B-RP

##### Genetic Features

*PDE6B* encodes the beta-subunit of the rod cGMP-phosphodiesterase, an enzyme that plays a key role during phototransduction. Mutations in *PDE6B* cause 2–5% of autosomal recessive retinitis pigmentosa (arRP) and rarely AD congenital stationary night blindness (CSNB) (181, 182).

##### Gene Therapy

After preclinical evidence that intraocular administration of the normal *PDE6B* gene preserved retinal morphology and functions in a mouse model of RP, a phase 1/2 clinical trial for subretinal administration of AAV2/5-hPDE6B was recently initiated (NCT03328130).

#### PDE6A-RP

##### Genetic Features

*PDE6A* encodes the alpha-subunit of the rod cGMP-phosphodiesterase. The loss of this enzyme function leads to chronically elevated cGMP levels, which cause an increased calcium inflow into the cell and thereby the hyperactivation of cell death pathways. Mutations in *PDE6A* cause 2–5% of arRP (183).

##### Gene Therapy

Patients with biallelic mutations of the *PDE6A* genes usually exhibit a mild to moderate phenotype, with an elevated degree of symmetry between the two eyes and with a relatively slow diseases course, though most patients have constricted VF by their fourth decade of life (184). Considering these features, PDE6A-related RP stands out as a compelling candidate for those gene therapy approaches requiring viable rods.

At the end of 2020, a phase 1/2 clinical trial sponsored by STZ eyetrial was commence and is currently open to enrolment (NCT04611503).

#### RLBP1-RP

##### Genetic Features

*RLBP1* (also known as *CRALBP*) encodes cellular retinaldehyde-binding protein, which acts primarily as an acceptor of 11-cis retinal during the isomerization step of the visual cycle. Mutations in *RLBP1* can cause three early-onset forms of arRP: retinitis punctata albescens, characterized by round punctate white deposits scattered throughout the entire retina in young patients with progression to more severe phenotypes in older individuals, Newfoundland rod-COD and Bothnia dystrophy, the latter two associated with a more severe prognosis. *RLBP1* mutations can also cause fundus albipunctatus, which is considered as a subtype of CSNB (185–188).

##### Gene Therapy

Proof of concept of the efficacy of gene replacement in *RLBP1*-related RP came from a study conducted on a mouse model of the disease, in which self-complementary AAV8 vector carrying the gene for human RLBP1 under control of a short RLBP1 promoter (scAAV8-pRLBP1-hRLBP1, or CPK850) was delivered via SRI, resulting in an improved electroretinographic response (189). The success of the preclinical study, followed by the publication of non-clinical safety data by the same group of authors (190), paved the way to clinical trials. To date, there is one ongoing phase 1/2 trial opened for recruiting, aimed at exploring the maximum tolerated dose, safety, and potential efficacy of CPK850 delivered through a single SRI. The trial is scheduled to end in 2026.

#### MERTK-RP

##### Genetic Features

*MERTK* encodes the widely expressed tyrosine-protein kinase Mer, a receptor tyrosine kinase involved in a signal transduction pathway that regulates numerous cellular processes. In the retina, it is expressed in the RPE and it is involved in the phagocytosis of rod OSs. *MERTK* mutations are responsible for arRP, with onset within the second decade of life and progressive decline of VA, which is often reduced to light perception before age 50 (191, 192).

##### Gene Therapy

An open-label, dose escalation phase 1 trial of AAV2-mediated gene augmentation therapy for RP caused by *MERTK* mutation was conducted in Saudi Arabia (193). The SRI of the vector was not associated with major side effects, and 50% of patients (three out of six) demonstrated improved VA, though only one of them maintained this improvement after 2 years of follow-up.

### Gene Therapy in Usher Syndrome (RP)

**Figure 2:**
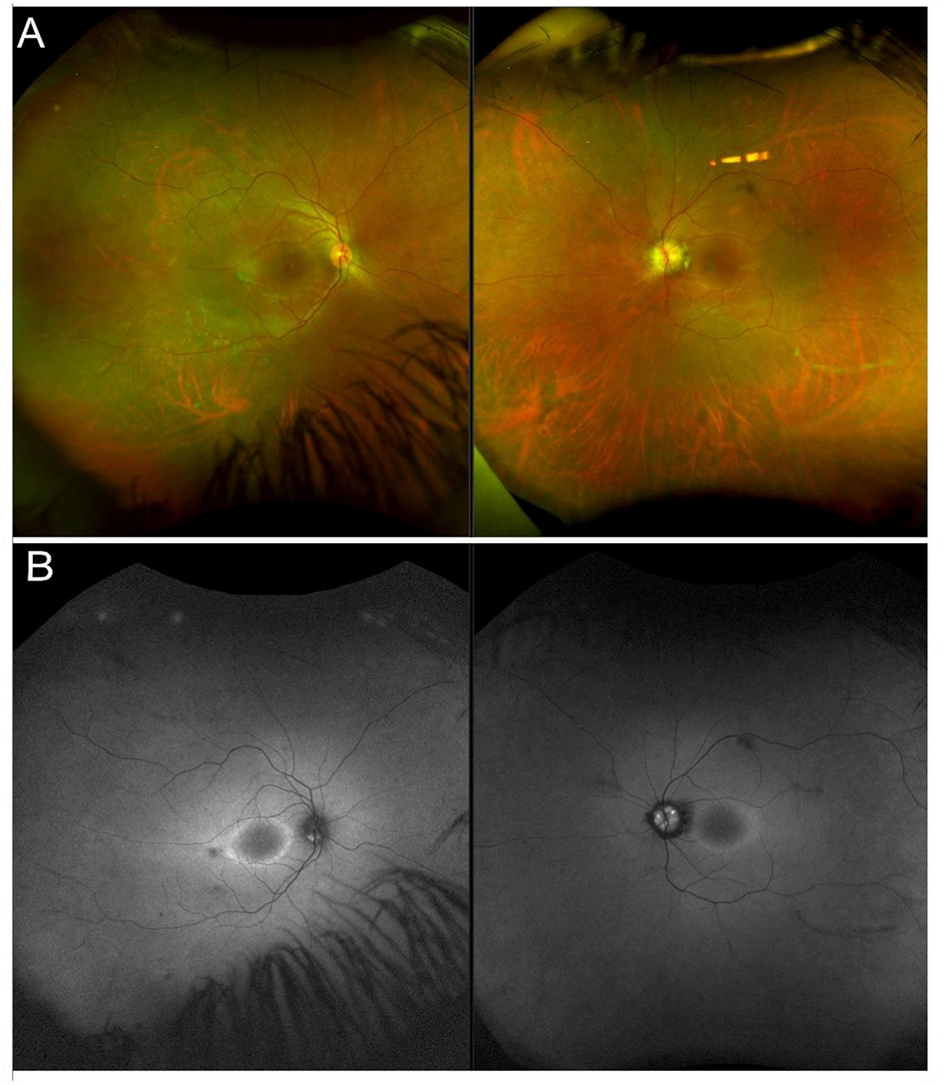
Multimodal imaging in a patient with a non-syndromic USH2A-related form of RP. **(A)** Ultra-wide field retinography shows near complete absence of pigment bone spicules. **(B)** Fundus autofluorescence displays the typical perifoveal ring of hyperautofluorescence.

Usher syndrome is defined by the association of AR deafness (most commonly congenital) and retinopathy indistinguishable from typical RP (Figure 2). Usher syndrome is the most common RP-associated syndrome, accounting for almost 20% of all RP patients. Depending on the severity of the hearing loss, Usher syndrome is divided into three clinical subtypes (194).

Usher syndrome type 1 (the most common form) is characterized by profound, congenital sensorineural deafness (with consequent prelingual deafness or severe speech impairment), vestibular symptoms and childhood onset retinopathy.

Usher syndrome type 2 presents with congenital partial, non-progressive deafness, absence of vestibular symptoms, and milder and later-onset retinopathy.

Finally, Usher syndrome type 3 is characterized by progressive deafness starting in the second to fourth decade, adult-onset retinopathy and hypermetropic astigmatism.

#### Genetic Features

To date, 16 genes associated to Usher syndrome have been identified, two of which are good candidates for gene therapy and deserve a more detailed description.

The first of such genes is *MYO7A*. *MYO7A* encodes myosin VIIA, involved in in transport of melanosomes and phagosomes along actin filaments in the RPE and of opsin and other phototransduction proteins in photoreceptors (195). Mutations in *MYO7A* are associated with Usher Syndrome type 1B (*USH1B*).

The second relevant gene is *USH2A*, which encodes usherin. Mutations in *USH2A*, besides being the commonest association with type 2 Usher syndrome (80%), are the most frequent cause of AR non-syndromic RP (10–15%) (196–199). Clear genotype-phenotype correlations for *USH2A* mutations are not easy to establish. Generally, however, nonsense mutations, frameshifts mutations, or canonical splice site mutations in *USH2A*, either biallelic or combined with one missense allele, are associated with Usher syndrome type II, whereas the association of two missense mutations tends to result in non-syndromic RP (200). Of note, a peculiar feature of both non-syndromic and syndromic *USH2A*-related retinopathy is the fact that fundoscopy generally shows mild or no pigment deposits.

#### Gene Therapy

The MYO7A gene has 49 exons and spans approximately 87 kb of genomic sequence on chromosome 11q13.5, therefore significantly exceeding the cargo capacity of AAV vectors. For this reason, attempts have been made to deliver *MYO7A* in the *shaker1* mouse model of Usher syndrome type 1B by means of UshStat, an EIAV lentiviral vector carrying the wild-type gene (EIAV-CMV-MYO7A) (201). Later on, Sanofi sponsored two phase 1/2 clinical trials, one of which is currently ongoing (NCT02065011), while the other has been stopped not for safety reasons (NCT01505062).

As far as *USH2A* is concerned, though being associated with recessive IRDs, a number of factors, including poor understanding of the physiological function of usherin and the *USH2A* gene size (15 kb), stand in the way of gene replacement strategies. Therefore, in order to develop an alternative approach, great interest was focused on a subset of mutations on exon 13 resulting in aberrant pre-mRNA splicing, which leads to the inclusion of a pseudoexon in the mature *USH2A* transcript. Since exon 13 consists of a multiplier of three nucleotides, skipping this exon does not disturb the open reading frame and likely results in the synthesis of a shorter protein with predicted residual function. This is the rationale behind the employment of AONs in an AR IRD. Encouraging results came from preclinical studies (73, 202) and ProQR Therapeutics sponsored the STELLAR phase 1/2 clinical trial (NCT03780257), whose purpose is to evaluate the safety and tolerability of a single intravitreal administration of AONs (QR-421a) in subjects with RP due to mutations in exon 13 of the *USH2A* gene. Enrolled patients receive one single IVT injection of QR-421a or sham-procedure in one eye (subject's worse eye) and are then followed up for 24 months. In March 2021, ProQR announced positive results from clinical trial of QR-421a and planned to start two phase 2/3 trials (SIRIUS and CELESTE).

### Gene Therapy in Choroideremia

**Figure 3:**
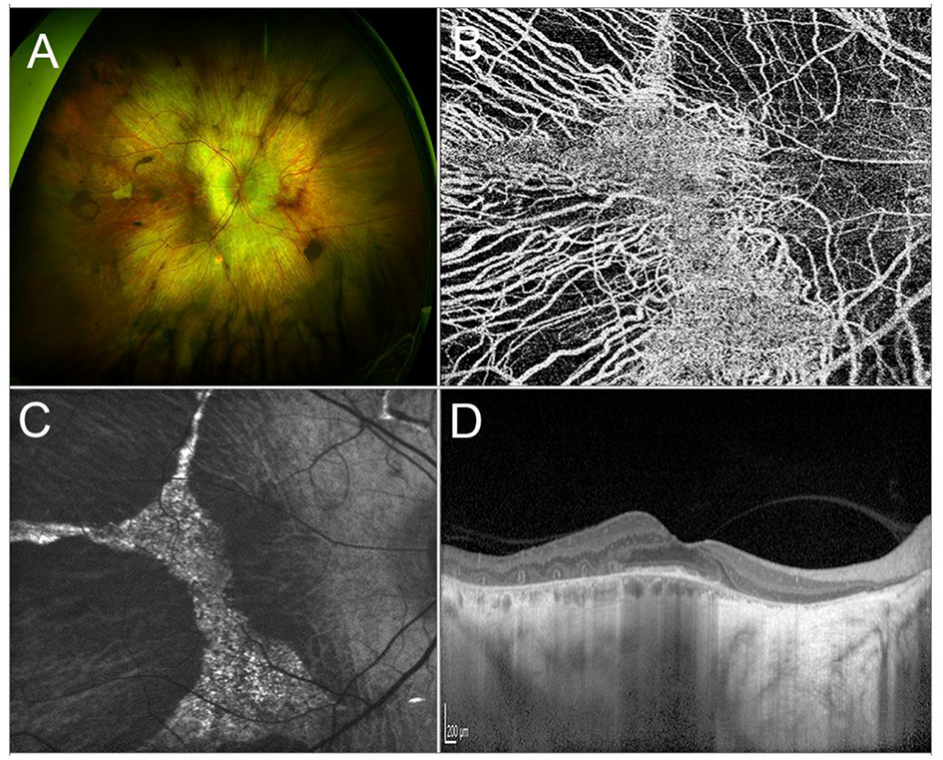
Multimodal imaging in CHM. **(A)** Ultra-wide field retinography shows a widespread chorioretinal atrophy, with a central islet of retinal sparing. **(B)** OCTA detects the sparing of the choriocapillaris vascular layer limited to the central islet. **(C)** Fundus autofluorescence displaying a central hyperautofluorescent region surrounded by hyporeflective atrophic tissue. **(D)** Structural OCT scan passing shows chorioretinal atrophy and outer retinal tubulations in the parafovea and a relative sparing of the photoreceptor layer in the fovea.

Choroideremia (CHM) is a rare XLR IRD, characterized by a progressive, centripetal, retinal degeneration, with a prevalence of 1:50,000 cases (203, 204). Vision loss progresses from nyctalopia in childhood to VF constriction in early adulthood and, ultimately, to legal blindness by the fifth decade of life (205, 206). From a pathogenic standpoint, Müller cells are the site of first damage, which is followed by outer retinal degeneration and, finally, by inner retinal thinning (207). Multimodal imaging findings include extended retinal hypoautofluorescence, with exclusive sparing of a central islet that allows patients to retain a good central vision. Structural OCT outside of this central region usually shows complete outer retinal atrophy (i.e., involving both photoreceptors and RPE cells), outer retinal tubulations and thinning of inner retinal layers and choroid (208, 209). Optical coherence tomography angiography displays an almost preserved superficial capillary plexus and choriocapillaris, with a significantly compromised deep capillary plexus (DCP), in the central islet, surrounded by completely absent choriocapillaris in the rest of the retina (210) (Figure 3).

#### Genetic Features

Choroideremia is caused by mutations in the *CHM* gene, which encodes component A of Rab geranylgeranyl-transferase, referred to as Rab escort protein 1 (REP1), a key mediator of post-translational lipidation (prenylation) and subcellular localization of a family of intracellular protein trafficking regulators, known as the Rab GTPases (211). The *CHM* gene is ubiquitously expressed, but in most tissues, including adrenal gland, brain, and thyroid, the homolog REP2 protein partially counterbalances REP1 deficiency. The reasons why REP2 does not prevent disease manifestation in the eye are yet to be elucidated. In rare instances, CHM may be part of a contiguous gene syndrome involving Xq21. Indeed, males with large interstitial deletions of an additional X-chromosome portion, other than Xq21, may develop CHM together with birth defects (cleft lip and palate and agenesis of the corpus callosum) and severe cognitive deficits (212). Moreover, previous reports described cases of males with a small deletion of Xq21 presenting with CHM, mixed sensorineural and conductive hearing deficits (in case of deletion of *POU3F4*), and varying degrees of cognitive deficits (in case of deletion of *RSK4*) (213). In addition, a previous report described the case of a female patient affected by CHM, sensorineural deafness, and primary ovarian failure secondary to a balanced X-4 translocation (214). A large study, involving more than 70 patients affected by this disease, reported a 94% rate of identification of a *CHM* mutation (204). Mutations of uncertain significance included non-contiguous duplications, insertion, deletion, point mutations, and aberrant splicing (204). It is worth of notice the absence of disease-causing missense mutations, in contrast to the majority of human genetic diseases, which are mainly determined by such mutations (204). To date, no defined genotype–phenotype correlation has been identified for CHM.

#### Gene Therapy

Choroideremia is a promising candidate for gene therapy since the 1.9 kb CHM cDNA is small enough to fit the size capacity of AAV vectors. Preclinical proof-of-concept studies on the feasibility of CHM gene replacement have been conducted both *in vitro*, by inducing the CHM gene in pluripotent stem cells (iPSCs) from patients with CHM, and *in vivo*, by delivering the AAV2-CHM virus in normal sighted mice and zebrafish, with no evidence of toxicity (215, 216), paving the way to clinical trials on CHM patients. The results of the first-in-human clinical trial date back to 2014, when MacLaren et al. reported phase I safety and efficacy data on six patients treated with low-dose subretinal AAV2-REP administered subfoveally, demonstrating an improvement in BCVA and retinal sensitivity for up to 3.5 years after treatment (217). These findings were confirmed by the 24-month data coming from the phase II trial (218). Less encouraging results came from another phase I clinical trial using the same vector at higher doses in six patients, since one of the six untreated eyes exhibited an improvement of >15 ETDRS letters, prompting the authors to conclude that VA should not be used as a primary outcome measure for future CHM gene therapy trials (219). So far, several clinical trials employing SRI of AAV2.REP1 have been conducted (NCT02671539, NCT02077361, NCT02553135, NCT02341807, NCT03496012). Combining together their results, overall 40 patients have been treated with a median gain of 1.5 ±7.2 SD in ETDRS letters, highly variable between the different trials (220). Although some issues still need to be addresses, such as the identification of proper endpoints and the development of safety enhancements to facilitate subretinal gene delivery, gene therapy for CHM has reached phase III clinical trials, providing real promise for patients. It is worth of notice the launch of the first phase I CHM clinical trial aimed at evaluating safety, tolerability, and preliminary efficacy of a single IVI of a rAAV gene therapy, 4D-110, in male patients with genetically confirmed CHM (NCT04483440).

### Gene Therapy in X-Linked Retinoschisis

**Figure 4:**
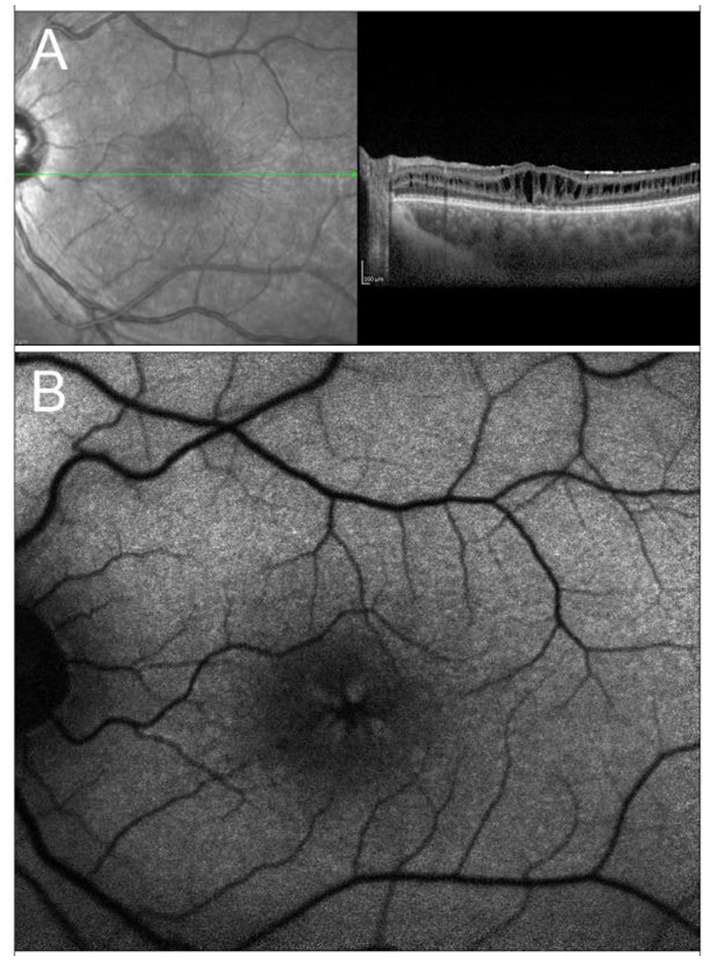
Multimodal imaging in X-linked retinoschisis. **(A)** Combined infrared reflectance imaging and structural OCT. Note the spokewheel pattern of folds radiating out from the fovea, which corresponds to the foveal schitic cavities seen on structural OCT. **(B)** Fundus autofluorescence also shows the spokewheel pattern due to the overlying foveal schisis.

X-linked retinoschisis is an IRD caused by mutations in the *RS1* gene on Xp22.1. With an estimated prevalence ranging between 1 in 15,000 and 1 in 30,000, it is the most common form of juvenile-onset retinal degeneration in males, whereas heterozygous female carriers usually do not display any symptoms (221, 222). From a clinical standpoint, the typical feature of the disease, present in 98–100% of cases, is the foveal schisis, often seen as a spokewheel pattern of folds radiating out from the fovea, with peripheral retinoschisis being encountered in about 50% of patients (223–225) (Figure 4). Visual acuity generally starts declining in the first two decades of life, followed by a very slow progression of macular atrophy until the fifth or sixth decade, with possible evolution to legal blindness (226, 227). However, patients may also have a better prognosis, as long as the most common complications (i.e., retinal detachment and vitreous hemorrhage) do not occur.

Electroretinography is helpful in the diagnosis of XLRS, since there is a typically reduced b-wave amplitude, with a relatively preserved a-wave amplitude (the so-called “negative” waveform) (228).

Multimodal imaging findings include visualization of macular schisis on structural OCT and detection of foveal vascular impairment at the DCP level upon OCTA (229).

#### Genetic Features

*RS1* encodes retinoschisin, a secretory protein exclusively expressed in retinal photoreceptors and bipolar cells, that can however be detected in all neuroretinal layers (230–232). Retinoschisin is found in a homo-oligomeric forms and, more specifically, it is an octamer made up of eight identical discoidin domains joined by intramolecular disulphide bonds (233). Mutations in the *RS1* sequence disrupt subunit assembly, thus interfering with retinoschin's role in retinal cell adhesion and organization of retinal architecture (233, 234).

#### Gene Therapy

Preclinical studies have shown that IVI administration of AAV8-scRS/IBPhRS vector, as well as SRI of the AAV5-mOPs-RS1 resulted in significant morpho-functional improvement in the retinoschisin knockout (*Rs1-KO*) mouse, with evidence of good tolerability in rabbits (235–238). Building upon these encouraging results, two phase 1/2 trials were initiated with different constructs, administered via IVI, since XLRS is an inner retinal pathology. For the first of such trials (NCT02317887), sponsored by the National Eye Institute (NEI), employing AAV8-scRS/IRBPhRS in adults (≥18 years old), promising initial findings have already been reported (239). The second trial (NCT02416622) uses rAAV2tYF-CHhRS1, intravitreally injected in adults (≥18 years old) in the first dose-escalation phase, with subsequent enrolment of individuals ≥6 years of age, after the maximum tolerated dose is identified.

### Gene Therapy in Stargardt Disease

**Figure 5:**
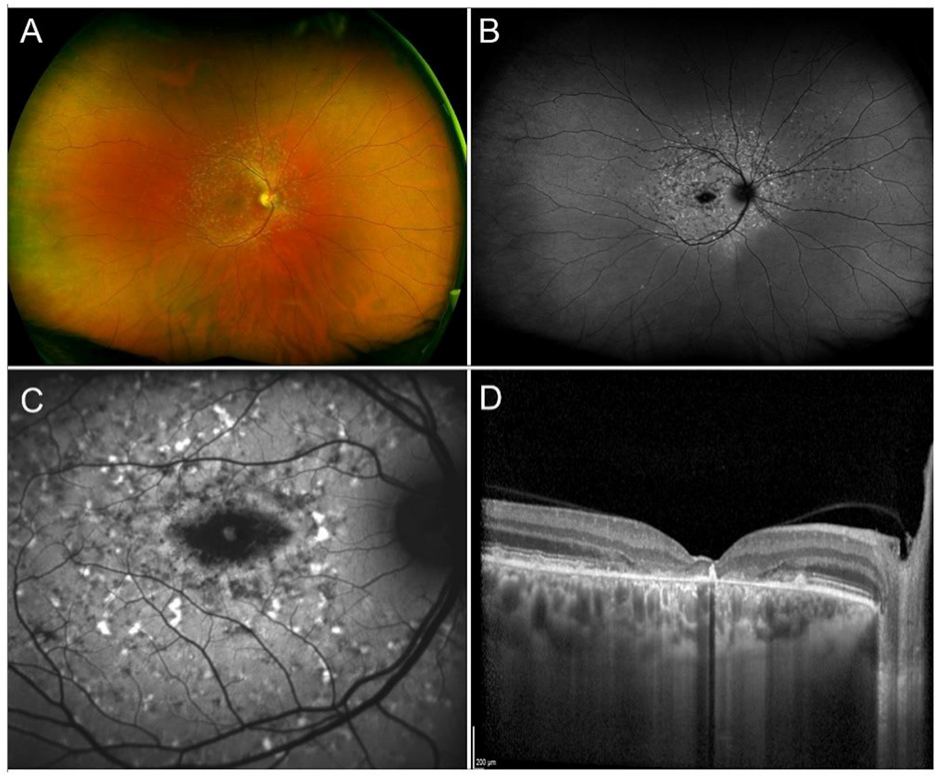
Multimodal imaging in Stargardt disease. **(A)** Ultra-wide field retinography shows numerous flecks inside and outside of the vascular arcades. **(B)** Ultra-wide field fundus autofluorescence displays the flecks as mixed hypo- and hyper-autofluorescent dots and shows an area of central atrophy, better highlighted by 30° fundus autofluorescence **(C)**. **(D)** Structural OCT shows chorioretinal atrophy and clumping of hyperreflective lipofuscin-rich material corresponding to flecks.

Stargardt disease, the most common hereditary macular dystrophy, is characterized by a progressive, centrifugal, macular degeneration, associated with different patterns of peripheral retinal alterations (240). The prevalence is of 1:8,000–10,000 individuals (240). From a clinical standpoint, one of the most typical findings in STGD are flecks, namely debris accumulations resulting from the progressive degeneration of RPE cells. Flecks have been described as hyperautofluorescent, corresponding to lipofuscin accumulations, and hypoautofluorescent, resulting from debris absorption and outer retinal atrophy onset (241). Other fundus findings include complete hypoautofluorescent central atrophy, surrounded by a halo of patchy, mottled hypoautofluorescence (242) (Figure 5). More recently, different STGD patterns have been described, characterized by progressively wider involvement of mid and extreme retinal periphery, as assessed by ultrawide field imaging (243). Furthermore, quantitative multimodal imaging and OCTA allowed to categorize STGD eyes accordingly to the amount of involvement of the retinal vascular and choroidal networks, highlighting different morpho-functional features and progression rates (156, 244, 245).

#### Genetic Features

Stargardt disease can be distinguished in three different forms: (I) STGD1, the most common form, displays an AR homozygous or compound heterozygous transmission and is caused by mutations in the *ABCA4* gene; (II) STGD3, which is determined by AD mutations in the *ELOVL4* gene; (III) STGD4, a rare AD form associated with mutations in the *PROM1* gene.

*ABCA4*, whose mutations are responsible for STGD1, is a 150 kb gene encoding an ATP-binding cassette transporter localized along the rims of photoreceptor OSs (246). This ATP-dependent flippase importer transports phosphatidylethanolamine (PE) and the all-*trans*-retinal (atRAL)/PE Schiff base (*N*-Ret-PE) into the cytosol, where atRAL is converted to all-*trans*-retinol (atROL) by retinol dehydrogenase RDH8 and RDH12. The absence of ABCA4 results in the accumulation of photo-toxic bisretinoids (A2E) with lipofuscin buildup in the RPE.

STGD3-causing *ELOVL4* gene contains six exons and plays a fundamental role in the synthesis of very long-chain polyunsaturated fatty acids (247).

Finally, the *PROM1* gene contains 23 exons distributed within a genomic sequence of more than 50 kb and encodes a protein involved in the organization of the plasma membrane and in the biogenesis of photoreceptor disks (248).

Genotype-phenotype correlations are challenging due to the heterogeneous disease manifestations. The most common genotypic classification includes three groups: genotype A (carriers of two or more deleterious variants); genotype B (one deleterious variant and >1 missense or in-frame insertion/deletion variants); genotype C (two or more missense or in-frame insertion or null variants) (249). Previous investigations highlighted how deleterious variants tend to be associated with more aggressive forms of the disease, while missense mutations yield to milder phenotypes (249–252). On the other hand, null alleles result in more severe STGD forms with an earlier onset (253). Other causes of severe phenotypes include truncating and severely misfolding mutations, deletions, stop codons, and insertions (254, 255). Furthermore, hypomorphic and deep intronic variants influencing the splicing process, have been also described (72, 256–258).

#### Gene Therapy

The main obstacles to the development of gene therapy approaches for STGD regard the dimension of the *ABCA4* gene, whose coding sequence exceeds the cargo capacity of AAV, and the extreme complexity of deleterious variants, as previously described.

In the wake of the success of animal studies using lentiviral gene therapy to deliver the corrected *ABCA4* gene (259), starting from 2011, Sanofi sponsored a phase I/II clinical trial (NCT01367444) to test the efficacy of a SRI of SAR422459, a recombinant lentiviral vector (EIAV) transporting a modified form of the *ABCA4* gene. Notwithstanding the encouraging preliminary findings, in 2020, the sponsor decided to stop development of the product for non-safety reasons.

In 2019, the Applied Genetic Technologies Corporation announced the development of a hybrid AAV dual vector and published preclinical data supporting the potential of this technology in STGD (260).

More recently, promising results have been reported in animal models with a non-viral technique relying on subretinal delivery of self-assembled NPs (261, 262). Compared to viral vectors, non-viral delivery systems have unlimited payload, low immunogenicity, and minimal side effects, features that may allow to circumvent the obstacles which are currently standing in the way of STGD gene therapy (263).

### Gene Therapy in Achromatopsia

Achromatopsia (ACHM) is an AR cone dysfunction affecting approximately 1 in 30,000 individuals (264, 265). Achromatopsia is a phenotypically and genotypically heterogeneous disease that can present in a complete or incomplete form. Complete ACHM is characterized by a totally abolished cone function, with a BCVA that is usually no >20/200 and with a total absence of color perception. In incomplete ACHM, residual cone function is present and patients have a higher VA and some degree of color discrimination (266). Prominent features of both complete and incomplete ACHM include photophobia, pendular nystagmus, central scotomata and high refractive (usually hypermetropic) errors (266). Electroretinography shows non-recordable cone-mediated responses with normal o near-normal rod responses (267). Fundus examination is normal or features non-specific alterations, such as central pigment mottling or attenuation of foveal reflex (268). On OCT, ACHM patients may display a variable phenotype, ranging from a normal to disrupted or absent EZ, which can sometimes be replaced by a hyporeflective cavitation, to complete outer retinal atrophy including the RPE (269). Fundus autofluorescence can be normal or show hyperautofluorescence, usually in zones of preserved EZ and likely preceding photoreceptor loss, or hypoautofluorescence, localized to areas of photoreceptor loss or RPE atrophy (270). Interestingly, adaptive optics scanning laser ophthalmoscopy (AOSLO) has shown the presence of cones in all ACHM patients, albeit reduced in number and with a highly variable density, regardless of OCT appearance, including areas of absent EZ reflectivity (271). This has important applications when it comes to patient selection for gene therapy trials.

#### Genetic Features

Mutations in six genes are responsible for over 90% of all ACHM cases, five of which are involved in the phototransduction process.

*CNGA3* and *CNGB3*, encoding for the a- and b-subunit of the cyclic nucleotide-gated (CNG) cation channel 3 found in cones OSs, together account for 70–80% of cases of ACHM worldwide (272).

*GNAT2* encodes the catalytic a-subunit of the G-protein transducin and is responsible for an infrequent form (<2%) of ACHM (273).

Equally rare subtypes are those caused by mutations in the *PDE6C* and *PDE6H* gene, encoding for the catalytic a- and inhibitory g-subunits of the photoreceptor-specific phosphodiesterase (274, 275).

Most recently, *ATF6* has been identified as a sixth ACHM-associated gene. ATF6 encodes a transmembrane TFs ubiquitously expressed and involved in endoplasmic reticulum homeostasis (276). The frequent finding of foveal hypoplasia in ATF6-ACHM lead to the suggestion that this gene may be crucial for foveal development (276).

#### Gene Therapy

Promising preclinical results have been reported for ACHM caused by mutations in the *CNGA3, CNB3*, and *GNAT2* genes. Taken together, these studies, which have been conducted on knock-out mouse models and naturally occurring mouse, sheep, and canine models, suggest that gene replacement approaches are effective and durable in ACHM, especially if administered early in life (277–284).

Other than for these encouraging data, ACHM seems particularly suited for gene augmentation for a number of reasons, including the presence of viable cones in all patients, as demonstrated by means of AOSLO, and its stationary or slowly progressive nature, which provides a wide window of opportunity.

The first phase I/II clinical trial commenced in November 2015 (NCT02610582) in Germany to assess the safety and efficacy of SRI of rAAV.hCNGA3 in patients with CNGA3-ACHM, using a dose-escalation protocol. Short after, another phase I/II clinical trial for CNGA-related ACHM (NCT02935517) was initiated in the US and in Israel and is still ongoing.

As far as CNGB3-ACHM is concerned, a phase I/II dose-escalation trial has been conducted in the UK (NCT03001310) to test the efficacy and safety of subretinal delivery of AAV2/8-hCARp.hCNGB3 and another similar multicentric phase I/II trial is still ongoing in the US and in Israel (NCT02599922).

### Final Remarks

In the present review, we tried to resume all the relevant findings and the present status of gene therapy in IRDs. On the basis of the above-described data (all the quoted clinical trials are listed in Table 2), a clinically applicable gene therapy represents a tangible perspective more than a still far target. Some IRDs seem to be closer to an upcoming definitive gene therapy treatment, whereas further studies are warranted for other ones. Overall considering all the techniques and approaches under investigation, the main current limitations include the safety profile of gene therapy, especially regarding the surgically-related risks for the retina, and sometimes the need of repeated treatments. The intravitreal route of administration might provide higher safety and feasibility profiles, although limiting the penetration of the treatment and drug concentrations effectively reaching retinal targets, if compared with subretinal approaches, turning out to be powerful but potentially riskier. Furthermore, it is known that each IRD may be characterized by extremely heterogeneous genotypic-phenotypic relationship. This is quite challenging to be evaluated both in clinical practice and in research contexts. We may assume that the different phenotypic expression of the mutated gene might have an influence not only on the morpho-functional status of the patients, but also on the clinical effect of gene therapy. From this point of view, future prospective studies should be focused on deeper assessments of genotypic-phenotypic features of each IRD, on new classification strategies and on the meanings that these advances in knowledge might have on gene therapy planning.

**Table 2:** Clinical trials on IRDs.

**N**	**Trial ID**	**Study title**	**Disease**	**Drug**	**Start date**	**Stop date**	**Phase**	**Status**
1	NCT03913143	Double-masked, randomized, controlled, multiple-dose study to evaluate efficacy, safety, tolerability, and syst. exposure of QR-110 in Leber's congenital amaurosis (LCA) due to C.2991+1655A>G mutation (p.Cys998X) in the CEP290 gene	Leber congenital amaurosis	Sepofarsen (QR-110)	2019	Ongoing	Phase I/II	Active, not recruiting
2	NCT02556736	Phase I/IIA, open-label, dose-escalation study of safety, and tolerability of intravitreal RST-001 in patients with advanced retinitis pigmentosa (RP)	Retinitis pigmentosa	RST-001	2015	Ongoing	Phase I/II	Active, not recruiting
3	NCT03326336	A phase 1/2a, open-label, non-randomized, dose-escalation study to evaluate the safety, and tolerability of GS030 in subjects with retinitis pigmentosa	Retinitis pigmentosa	GS030-DP	2017	Ongoing	Phase I/II	Recruiting
4	NCT04278131	Phase 1/2, safety, and efficacy trial of BS01, a recombinant adeno-associated virus vector expressing chronosFP in patients with retinitis pigmentosa	Retinitis pigmentosa	BS01	2020	Ongoing	Phase I/II	Recruiting
5	NCT04919473	A phase I/IIa open label, dose-escalation study to evaluate the safety, and tolerability of intravitreal vMCO-I in patients with advanced retinitis pigmentosa	Retinitis pigmentosa	vMCO-I	2019	2020	Phase I/II	Completed
6	NCT03602820	A long-term follow-up study in subjects who received an adenovirus-associated viral vector serotype 2 containing the human RPE65 gene (AAV2-hRPE65v2, voretigene neparvovec-rzyl) administered via subretinal injection	Leber congenital amaurosis	AAV2-hRPE65v2	2015	Ongoing	N/A	Active, not recruiting
7	NCT01208389	A follow-on study to evaluate the safety of re-administration of adeno-associated viral vector containing the gene for human RPE65 [AAV2-hRPE65v2] to the contralateral eye in subjects with leber congenital amaurosis (LCA) previously enrolled in a phase 1 study	Leber congenital amaurosis	AAV2-hRPE65v2	2010	Ongoing	Phase I/II	Active, not recruiting
8	NCT03913143	Double-masked, randomized, controlled, multiple-dose study to evaluate efficacy, safety, tolerability, and syst. exposure of QR-110 in Leber's congenital amaurosis (LCA) due to c.2991+1655A>G mutation (p.Cys998X) in the CEP290 gene	Leber Congenital Amaurosis	Sepofarsen (QR-110)	2019	Ongoing	Phase II/III	Active, not recruiting
9	NCT03116113	A dose escalation (phase 1), and dose expansion (phase 2/3) clinical trial of retinal gene therapy for X-linked retinitis pigmentosa using an adeno-associated viral vector (AAV8) encoding retinitis pigmentosa GTPase regulator (RPGR)	Retinitis pigmentosa	BIIB112	2017	2020	Phase I/II	Completed
10	NCT03252847	An open label, multi-center, phase I/II dose escalation trial of a recombinant adeno-associated virus vector (AAV2-.RPGR) for gene therapy of adults and children with X-linked retinitis pigmentosa owing to defects in retinitis pigmentosa GTPase regulator (RPGR)	Retinitis pigmentosa	AAV2/5-RPGR	2017	Ongoing	Phase I/II	Active, not recruiting
11	NCT04671433	Phase 3 randomized, controlled study of AAV5-RPGR for the treatment of X-linked retinitis pigmentosa associated with variants in the RPGR gene	Retinitis pigmentosa	AAV5-RPGR	2021	Ongoing	Phase III	Recruiting
12	NCT03316560	A phase 1/2 open-label dose escalation study to evaluate the safety and efficacy of AGTC-501 (rAAV2tYF-GRK1-RPGR) and a phase 2 randomized, controlled, masked, multi-center study comparing two doses of AGTC-501 in male subjects with x-linked retinitis pigmentosa confirmed by a pathogenic variant in the RPGR gene	Retinitis pigmentosa	rAAV2tYF-GRK1-RPGR	2018	Ongoing	Phase I/II	Recruiting
13	NCT04850118	A phase 2/3, randomized, controlled, masked, multi-center study to evaluate the efficacy, safety, and tolerability of two doses of AGTC-501, a Recombinant adeno-associated virus vector expressing RPGR (rAAV2tYF-GRK1-RPGR), compared to an untreated control group in male subjects with X-linked Retinitis pigmentosa confirmed by a pathogenic variant in the RPGR gene	Retinitis pigmentosa	rAAV2tYF-GRK1-hRPGRco	2021	Ongoing	Phase II/III	Not yet recruiting
14	NCT04517149	An open-label, phase 1/2 trial of gene therapy 4D-125 in males with X-linked retinitis pigmentosa (XLRP) caused by mutations in the RPGR gene	Retinitis pigmentosa	4D-125	2020	Ongoing	Phase I/II	Recruiting
15	NCT03328130	Safety and efficacy of a unilateral subretinal administration of HORA-PDE6B in patients with retinitis pigmentosa harboring mutations in the PDE6B gene leading to a defect in PDE6ß expression	Retinitis pigmentosa	AAV2/5-hPDE6B	2017	Ongoing	Phase I/II	Recruiting
16	NCT04611503	PIGMENT—PDE6A gene therapy for retinitis pigmentosa	Retinitis pigmentosa	rAAV.hPDE6A	2019	Ongoing	Phase I/II	Recruiting
17	NCT02065011	An open-label study to determine the long-term safety, tolerability, and biological activity of SAR421869 in patients with usher syndrome type 1B	Retinitis pigmentosa	SAR421869	2013	Ongoing	Phase I/II	Active, not recruiting
18	NCT01505062	A phase I/IIA dose escalation safety study of subretinally injected SAR421869, administered to patients with retinitis pigmentosa associated with usher syndrome type 1B	Retinitis pigmentosa	SAR421869	2012	2019	Phase I/II	Terminated for non-safety reasons
19	NCT03780257	A first-in-human study to evaluate the safety and tolerability of QR-421a in subjects with retinitis pigmentosa (RP) due to mutations in exon 13 of the USH2A gene	Retinitis Pigmentosa	QR-421a	2019	Ongoing	Phase I/II	Active, not recruiting
20	NCT02671539	THOR—tübingen choroideremia gene therapy trial open label phase 2 clinical trial using an adeno-associated viral vector (AAV2) encoding rab-escort protein 1 (REP1)	Choroideremia	rAAV2.REP1	2016	2018	Phase II	Completed
21	NCT02077361	An open label clinical trial of retinal gene therapy for choroideremia using an adeno-associated viral vector (AAV2) encoding Rab-escort protein-1 (REP1)	Choroideremia	rAAV2.REP1	2015	Ongoing	Phase I/II	Active, not recruiting
22	NCT02553135	An open label phase 2 clinical trial of retinal gene therapy for choroideremia using an adeno-associated viral vector (AAV2) encoding Rab-escort protein 1 (REP1)	Choroideremia	AAV2-REP1	2015	2018	Phase II	Completed
23	NCT02341807	A phase 1/2 safety study in subjects with CHM (choroideremia) gene mutations using an adeno-associated virus serotype 2 vector to deliver the normal human CHM gene [AAV2-hCHM] to the retina	Choroideremia	AAV2-hCHM	2015	Ongoing	Phase I/II	Active, not recruiting
24	NCT03496012	A randomized, open label, outcomes-assessor masked, prospective, parallel controlled group, phase 3 clinical trial of retinal gene therapy for choroideremia using an adeno-associated viral vector (AAV2) encoding Rab escort protein 1 (REP1)	Choroideremia	AAV2-REP1	2017	2020	Phase III	Completed
25	NCT04483440	Phase 1 Open-label, dose-escalation study of the safety, tolerability, and preliminary efficacy of intravitreal 4D-110 in patients with choroideremia	Choroideremia	4D-110	2020	Ongoing	Phase I	Recruiting
26	NCT02317887	A phase I/IIa study of RS1 ocular gene transfer for X-linked retinoschisis	X-linked retinoschisis	RS1 AAV	2015	Ongoing	Phase I/II	Recruiting
27	NCT02416622	A multiple-site, phase 1/2, safety, and efficacy trial of a recombinant adeno-associated virus vector expressing retinoschisin (rAAV2tYF-CB-hRS1) in patients with X-linked retinoschisis	X-linked retinoschisis	rAAV2tYF-CB-hRS1	2015	Ongoing	Phase I/II	Active, not recruiting
28	NCT01367444	A phase I/IIA dose escalation safety study of subretinally injected SAR422459, administered to patients with Stargardt's macular degeneration	Stargardt's disease	SAR422459	2011	2019	Phase I/II	Terminated for non-safety reasons
29	NCT02610582	Safety and efficacy of a bilateral single subretinal injection of rAAV.hCNGA3 in adult and minor patients with CNGA3-linked achromatopsia investigated in a randomized, wait list controlled, observer-masked trial	Achromatopsia	rAAV.hCNGA3	2015	Ongoing	Phase I/II	Recruiting
30	NCT02935517	A multiple-site, phase 1/2, safety, and efficacy trial of AGTC 402, a recombinant adeno-associated virus vector expressing CNGA3, in patients with congenital achromatopsia caused by mutations in the CNGA3 gene	Achromatopsia	AGTC-402	2017	Ongoing	Phase I/II	Recruiting
31	NCT03001310	An open label, multi-center, phase I/II dose escalation trial of a recombinant adeno-associated virus vector (AAV2/8-hCARp.hCNGB3) for gene therapy of adults and children with achromatopsia owing to defects in CNGB3	Achromatopsia	AAV-CNGB3	2017	2019	Phase I/II	Completed
32	NCT02599922	A multiple-site, phase 1/2, safety, and efficacy trial of a recombinant adeno-associated virus vector expressing CNGB3 in patients with congenital achromatopsia caused by mutations in the CNGB3 Gene	Achromatopsia	rAAV2tYF-PR1.7-hCNGB3	2016	Ongoing	Phase I/II	Recruiting

*The list follows the order of appearance in the manuscript (from https://clinicaltrials.gov/)*.

### Conclusions

Inherited retinal diseases are significantly disabling conditions affecting young, working-age populations. Despite the provision of low-vision aids and assistance from specialist services, to date the management of these disorders remains largely suboptimal and the development of definitive therapies should be regarded as a priority. Based on preclinical data and on an ever-growing body of clinical evidence, gene-based strategies can now be looked at with cautious optimism. However, whilst gene therapy holds great hope for the treatment of a wide range of IRDs in the future, there are caveats to be considered, which are mainly related to the careful selection of appropriate target diseases, patients, and outcome measures and to the surgical challenges of vector delivery. Natural history studies, long term follow-up of treated patients and advances in the field of genetic testing and molecular diagnostics are among the lines of research that can be pursued to address these issues and to expand the spectrum of IRDs that can be treated with this potentially revolutionary approach.

## Author Contributions

AAr and AAm: review design, data analysis, data interpretation, and manuscript draft. EA, AS, and MM: data acquisition and data analysis. MB and FB: data interpretation, manuscript revision, and study supervision.

## Conflict of Interest

FB consultant for Alcon (Fort Worth, Texas, USA), Alimera Sciences (Alpharetta, Georgia, USA), Allergan Inc. (Irvine, California, USA), Farmila-Thea (Clermont-Ferrand, France), Bayer Shering-Pharma (Berlin, Germany), Bausch and Lomb (Rochester, New York, USA), Genentech (San Francisco, California, USA), Hoffmann-La-Roche (Basel, Switzerland), NovagaliPharma (Évry, France), Novartis (Basel, Switzerland), Sanofi-Aventis (Paris, France), Thrombogenics (Heverlee, Belgium), and Zeiss (Dublin, USA). The remaining authors declare that the research was conducted in the absence of any commercial or financial relationships that could be construed as a potential conflict of interest.

## Publisher's Note

All claims expressed in this article are solely those of the authors and do not necessarily represent those of their affiliated organizations, or those of the publisher, the editors and the reviewers. Any product that may be evaluated in this article, or claim that may be made by its manufacturer, is not guaranteed or endorsed by the publisher.
